# Cortical morphological heterogeneity of schizophrenia and its relationship with glutamatergic receptor variations

**DOI:** 10.1192/j.eurpsy.2023.2408

**Published:** 2023-05-09

**Authors:** Xuan Ouyang, Yunzhi Pan, Xudong Chen, Guowei Wu, Yixin Cheng, Wenjian Tan, Manqi Zhang, Mengjie Deng, Zhening Liu, Lena Palaniyappan

**Affiliations:** 1 Department of Psychiatry, National Clinical Research Center for Mental Disorders, and National Center for Mental Disorders, The Second Xiangya Hospital of Central South University, Changsha, China; 2 Robarts Research Institute, Schulich School of Medicine and Dentistry, Western University, London, ON, Canada; 3 Department of Medical Biophysics, Schulich School of Medicine and Dentistry, Western University, London, ON, Canada; 4 Douglas Mental Health University Institute, Department of Psychiatry, McGill University, Montreal, QC, Canada

**Keywords:** Cortical folding, polymorphisms, prognosis, stratification, unsupervised machine learning

## Abstract

**Background:**

Recent genetic evidence implicates glutamatergic-receptor variations in schizophrenia. Glutamatergic excess during early life in people with schizophrenia may cause excitotoxicity and produce structural deficits in the brain. Cortical thickness and gyrification are reduced in schizophrenia, but only a subgroup of patients exhibits such structural deficits. We delineate the structural variations among unaffected siblings and patients with schizophrenia and study the role of key glutamate-receptor polymorphisms on these variations.

**Methods:**

Gaussian Mixture Model clustering was applied to the cortical thickness and gyrification data of 114 patients, 112 healthy controls, and 42 unaffected siblings to identify subgroups. The distribution of glutamate-receptor (GRM3, GRIN2A, and GRIA1) and voltage-gated calcium channel (CACNA1C) variations across the MRI-based subgroups was studied. The comparisons in clinical symptoms and cognition between patient subgroups were conducted.

**Results:**

We observed a “hypogyric,” “impoverished-thickness,” and “supra-normal” subgroups of patients, with higher negative symptom burden and poorer verbal fluency in the hypogyric subgroup and notable functional deterioration in the impoverished-thickness subgroup. Compared to healthy subjects, the hypogyric subgroup had significant GRIN2A and GRM3 variations, the impoverished-thickness subgroup had CACNA1C variations while the supra-normal group had no differences.

**Conclusions:**

Disrupted gyrification and thickness can be traced to the glutamatergic receptor and voltage-gated calcium channel dysfunction respectively in schizophrenia. This raises the question of whether MRI-based multimetric subtyping may be relevant for clinical trials of agents affecting the glutamatergic system.

## Introduction

Today, while we know much more about the pathophysiology of schizophrenia than we did more than a century ago when Bleuler described the construct of “a group of schizophrenias”, the recovery rates of this illness have not changed much[1–3]. As Bleuler suspected, converging evidence indicated schizophrenia to be a heterogeneous set, with no single biological underlying process that can be invoked to account for all of the diagnosed patients[4]. Nevertheless, we do not yet know how to distinguish one group of schizophrenia from another in an objective manner to enable us to advance in our mechanistic enquiries and for treatment selection––a key step to improve recovery rates in this condition.

In recent years, many studies have been conducted to dissect the heterogeneity of schizophrenia using measures of symptomatology[5, 6], neurocognition[7–9], genetics[10] and neuroimaging [11–14]. Among them, the use of brain morphology has been the most promising, and the most often replicated subtyping approach to date. Brain morphological features are more stable, with low within-subject fluctuations than symptom rating scores and functional readouts (fMRI, EEG). In addition, MRI-derived measures such as thickness and degree of cortical folding (gyrification) can be quantified objectively in an automatized manner with minimal manual intervention in the quantification process. Thus, brain structure can provide more stable and reliable clustering solutions. Among the various morphological indices, cortical thickness across multiple brain regions has been employed as a feature of interest in many subtyping studies[14–19], with 2-6 distinguishable subgroups reported across studies of schizophrenia[14–19]. While the number of subgroups varies among studies, likely due to inherent noise in clinical sampling and variations in analytical approaches, one consistent feature of these studies is the presence of a notable subgroup with widespread cortical thickness reduction (“impoverished cortex”) compared to healthy subjects[12, 14] and other patients[13, 20].

We have recently observed inappropriately high cortical glutamate levels in the subgroup with reduced thickness [13]. This raises the possibility of a persistent glutamatergic activation at the receptor level––affecting the N-methyl-d-aspartate acid (NMDA) and α-amino-3-hydroxy-5-methylisoxazole propionic acid (AMPA) receptors and voltage-gated calcium channels (VGCC)––leading to calcium overload, oxidative stress and excitotoxic damage to dendritic spines[21–23]. Excessive synaptic elimination with loss of dendritic spines is suspected to underwrite the MRI readout of reduced thickness[24]. This account is consistent with progressive cortical thinning noted in the early phase of psychosis[25, 26]. Nevertheless, it is important to note that ~80% of patients with schizophrenia do not show deviations (i.e., infra-normal levels) in thickness patterns[10, 27], while some may show supra-normal changes suggestive of adaptive or compensatory response[28, 29]. Leveraging the variance in thickness alone is unlikely to uncover the full spectrum of heterogeneity in schizophrenia.

Changes in synaptic density can reduce the MRI-based intensity of the grey matter, and affect MR-derived measures based on the location of the gray-white boundary (e.g., cortical thickness, volume). However, contour-based MRI measures such as the gyrification index (ratio of buried to outer cortex) are likely to be more stable in the face of grey matter loss. For example, models of progressive age-related brain atrophy estimate that while 75–164% increase in ventricular volume could occur in adults up to the age of 80, only 2.7% change may occur in the gyrification index. Furthermore, gyrification patterns emerge in utero, and change in response to intrauterine disruptions (e.g., hypoxia[30] and associated excitotoxicity[31]) but remain detectable in later life [27]. A large body of evidence now points to the presence of widespread reduction in gyrification (hypogyria) in established cases of schizophrenia [32–34], in conjunction with poor treatment response patterns [35] and other markers of aberrant neurodevelopment (e.g., Neurological Soft Signs [NSS]) [36, 37]. Thus, when clustering patients with schizophrenia using neuroanatomical information from multiple brain regions, employing gyrification index as a feature could identify a predominantly “hypogyric” subgroup with developmental aberrations, while thickness could identify a “reduced thickness” subgroup with excitotoxic tissue loss.

One of the major goals of a subtyping exercise in schizophrenia is to test if distinguishable biological mechanisms account for the presence of discrete subgroups of patients. Recently, the genome-wide association studies in schizophrenia have implicated various glutamate receptors and downstream calcium signaling pathways in this illness––such as GRM3[38], GRIN2A[39], GRIA1[40] and CACNA1C[41, 42] involved in the functional regulation of metabotropic glutamate receptors (mGluR), NMDA receptors, AMPA receptors, and VGCC. The glutamate receptor system and calcium signaling (regulated by CACNA1C) [43–46] are highly pertinent to the morphological changes in psychosis [47–50]. While the molecular mechanism influencing the generation of cortical thickness and gyrification are likely to be distinct [51], genetic variations in glutamate transmission could likely affect the development of both cortical gyrification (by influencing neuronal migration and subplate apoptosis [52–55]) and thickness (see Smith and Walsh for a review [56]). Furthermore, the ionotropic glutamate receptors (NMDA/AMPA [57] and VGCC [58] play a key role in regulating the glutamate-mediated excitotoxicity. In this context, we hypothesized that the genetic risk variants related to this process will be over-represented in the subgroups with reduced gyrification and thickness, but not in other patients with preserved cortical anatomy. To test this, we studied the polymorphisms pertaining to three main glutamatergic postsynaptic receptors: the NMDA, AMPA, mGluR, and the VGCC.

Morphological features, especially regional thickness, can be affected by several secondary features associated with schizophrenia, e.g., cannabis use, urban living, migration [59]. While polygenic risk for schizophrenia has a strong influence on the morphology [60, 61], the within-group variance among patients appears to reflect these secondary disease factors [62]. As a result, for subgroup-based genetic associations, leveraging within-group variance in healthy groups (especially unaffected siblings) will be important to uncover the suspected genetic associations in morphologically-driven subgroups.

In the current study, we estimate MRI-derived cortical thickness and gyrification across multiple brain regions in 3 groups: patients with schizophrenia, unaffected siblings of patients, and unrelated healthy individuals. Leveraging the within-group heterogeneity of cortical features among healthy as well as clinically and genetically affected groups [20, 63], we identify clusters of patients that have shared morphological features (within-cluster similarity), and study their clinical, cognitive, and genetic profiles (GRM3, GRIN2A, GRIA1, and CACNA1C variants). As cross-sectional phenotyping is insufficient to understand prognostic relevance, we followed up a consenting subsample (43 patients, average of 13.4 months) longitudinally and report prognostic associations. As an exploratory analysis, we also investigated if the cluster membership interacts with genetic polymorphisms in a region-specific manner to affect thickness and gyrification index. To foreshadow the result, we (1) confirm the existence of a “reduced thickness” and a “preserved cortex” subgroups, (2) report for the first time a distinct, predominantly hypogyric subgroup and (3) identify specific glutamate-receptor variations that may influence these anatomical phenotypes in schizophrenia.

## Methods and Materials

### Participants

Patients (SZ) (*n* = 114) with a diagnosis of schizophrenia (based on DSM-5 [64]) and their siblings (*n* = 42) were recruited from the inpatient and outpatient units at Second Xiangya Hospital of Central South University, Changsha, China from 2016 to 2021. The inclusion criteria of patients: (1) meet the DSM-5 diagnostic criteria for schizophrenia, and the diagnosis was later rechecked after 6 months through face-to-face or real-time video interview; (2) 12< age ≤ 35 years; (3) right-handed; and (4) normal intellectual development. The exclusion criteria included: (1) meet the diagnosis of any mental disorder(s) except schizophrenia in DSM-5; (2) any reported history of substance use, neurological disorder, or serious physical illness in themselves or their first-degree relatives; (3) any contraindication for MRI; (4) left-handedness (as China has a usually low prevalence of left-handedness, and exclusion was more practical than case-control matching) [65]; (5) history of brain injury or conscious coma; (6) intellectual disability (IQ<70) and (7) previous electroconvulsive therapy.

We also assessed the longitudinal change in symptoms and functional recovery in a subset of patients (*n* = 43) who were seeking help in a symptomatic state (81.4% with first-episode of illness, 4.7% for relapse, and 13.9% for persistent symptom burden after first episode) and were fully concordant with follow-up and received continuous antipsychotic treatment based on clinical records for 1-2 years. The average follow-up period was 13.4(±11.5) months. On initial presentation, 58.14% of the follow-up cohort had <1 month of lifetime exposure to antipsychotics; 25.58% of the patients had 3 to 12 months exposure; 16.28% of the patients have been treated for 12 to 30 months. This cohort was primarily recruited to assess subtype-specific differences in clinical and functional improvement rates.

In addition to patients and their siblings, healthy controls (HC) (*n* = 112) were recruited from the communities and schools in Changsha City. The inclusion criteria of HC are as below: (1) not meeting any diagnostic criteria for any mental disorders; (2) 12< age ≤ 35 years; (3) right-handed; and (4) normal intellectual development. The exclusion criteria were consistent with the criteria of schizophrenia patients, except for diagnosis. The authors assert that all procedures contributing to this work comply with the ethical standards of the relevant national and institutional committees on human experimentation and with the Helsinki Declaration of 1975, as revised in 2008. All participants gave written informed consent to the study approved by the local Ethics Committee of Second Xiangya Hospital.

### Targeted gene selection and sequencing

In this study, we selected the target regions in the genome to focus on the genes relevant to the receptors and channels on glutamate postsynaptic membrane. Briefly, we included GRM3 (involved in metabotropic glutamate receptors), GRIN2A (involved in NMDA ionotropic glutamate receptors), GRIA1 (involved in KA/AMPA ionotropic glutamate receptors), and CACNA1C (involved in voltage-gated calcium channel). For each of these genes, both coding and non-coding (regulatory) regions were included in the sequencing target. The regulatory genomic regions were comprised of 5’ un-translated region (5’ UTR), 3’ untranslated region (3’ UTR), and intron-exon boundaries (25bp). Custom capture oligos were designed using SureDesign website of Agilent Technologies (Santa Clara, CA)(https://earray.chem.agilent.com/suredesign/).

Blood samples of participants were collected on the day of MRI scan, and then genomic DNA was extracted for sequencing. Genomic DNA (2 μg) was used to target enrichment and to construct a DNA library before targeted sequencing. The genomic DNA was sheared to an average size of 250bp by using of Covaris S220 (Covaris, the USA), and the DNA library preparation and the capture procedure were then performed by using the SureSelect XT Target Enrichment System (Agilent, the USA), following the manual strictly. For all DNA libraries, the Illumina Hiseq2000 sequencing system (Illumina, San Diego, CA, USA) was employed to generate the paired-end 150bp reads raw data. Each sample was sequenced to >80% coverage at a minimum of 30-fold read depth. The Annovar program (dated 2016-02-01) was used for single nucleotide variation (SNV) annotation. Any SNV recorded in dbSNP147, with a minor allele frequency of ≥1% in 1000 genome database, ≥1% in our dataset, and with missing calls in <10% of subjects were considered as single nucleotide polymorphisms (SNP) and included for subsequent individual-variant association analysis (SNPs failing the Hardy-Weinberg equilibrium test at a significance level of 0.0001 were removed). The above bioinformatics analysis was described in Supplementary Material A.

### General data collection, cognitive test, and clinical assessment

For all participants, the general intelligence level was evaluated through Wechsler Adult Intelligence Scale (WAIS)-Digital symbol test[66], WAIS-Digit span test (Forward), WAIS-Digit span test (Backward), and WAIS-Arithmetic test. The social functioning level was evaluated through the Social and Occupational Functioning Assessment Scale (SOFAS) [67]. In terms of cognition, we adopted story retelling and N-back test[68] for memory function assessment, Verbal-Fluency test[69] for language function assessment, a visual pattern test[70] for visual perception assessment, and a Wisconsin card sorting test[71] for overall cognitive assessment.

For schizophrenia patients, the diagnoses were made by qualified psychiatrists according to DSM-5 criteria. On the same day as the MRI session, the severity of symptoms was evaluated through the Positive and Negative Syndrome Scale (PANSS) [72], the Scale for The Assessment of Positive Symptoms (SAPS) [73], the Scale for The Assessment of Negative Symptoms (SANS) [73], and the Schizophrenia Suicide Risk Scale (SSRS) [74]. The duration of illness, antipsychotic load (converted into chlorpromazine equivalent per day), and duration of antipsychotic medication were recorded. The PANSS, SAPS, SANS, and SOFAS of patients was assessed after at least 2 months of antipsychotic treatment. The rate of reduction in the scores of PANSS, SAPS, and SANS was calculated as (Baseline-Follow up)/ Baseline, and the improvement rate of SOFAS was calculated as (Follow up-Baseline)/Follow up. Thus, a positive value indicates a better outcome over time in both cases.

### Magnetic resonance image acquisition and image processing

The participants were scanned using a Siemens 3.0 Tesla MRI scanner at Second Xiangya Hospital of Central South University at Changsha, China. T1-weighted magnetic resonance imaging data were acquired using a three-dimensional spoiled gradient echo (SPGR) pulse sequence from the sagittal plane, scanning parameter as follow: TR=7.6 ms, TE=3.7 ms,FA =8°, 180 slices, matrix =256*200,and the field of view (FOV)=256×256 mm^2^,slices were contiguous with a slice thickness of 2 mm. Importantly, during the T1-weighted image acquisition, participants were asked to remain still, and if any motion-related artifacts were detected, the scans were repeated.

A surface-based approach using Free-Surfer (http://surfer.nmr.harvard.edu, version 7.1.1) was used to calculate the cortical thickness and gyrification in the whole brain. Following skull-stripping and intensity correction, the gray–white matter boundary for each cortical hemisphere was determined by tissue intensity and neighborhood constraints. The resulting surface boundary was tessellated to generate multiple vertices across the whole brain before inflating. Using a deformable surface algorithm guided by the gray–CSF intensity gradient, the resulting gray–white interface was expanded to create the pial surface. The inflated surface was then morphed into a sphere followed by registration to an average spherical surface for optimal sulcogyral alignment. Then, the vertex-wise method (advocated by Schaer et al. [75]) was used to continuously assess local gyrification index (LGI) of the entire cortex. This method is an extension of classical two-dimensional GI measurement that calculates the ratio of the pial perimeter over the outer perimeter on coronal sections [76]. It provides an LGI for each vertex on cortical surface, which reflects the amount of cortex buried in its immediate locality. After the above procedures, Desikan-Killiany Atlas (68 regions) was used to extract cortical thickness and gyrification of each region using the FreeSurfer software [77]. Topological defects were corrected manually by two members of the research staff via tktools of freesurfer (https://surfer.nmr.mgh.harvard.edu /fswiki/FsTutorial/TopologicalDefect_tktools).

### Statistical analysis

Clustering analysis was conducted in Matlab platform (version x). Before the clustering, the cortical thickness and gyrification of 68 regions were transformed to Z-scores. Then we used the clustering based on Gaussian Mixture Model (GMM) and GAP statistics to identify clusters of participants who shared similar patterns of cortical thickness and gyrification. Gaussian clustering was applied to all participants, including schizophrenia patients, unaffected siblings and HCs. We set cluster numbers from 1 to 6 (6 was the maximum value of the image structural subtype found so far) and GAP statistics to estimate the optimal number of clusters in our data. Then we chose the smallest cluster number that conformed to Gap(k) ≥ Gap(k+1) − S_k+1_ as the solution of cluster analysis based on the 1-standard error method suggested by Tibshirani [78].

One-way ANOVA (in SPSS 20.0) was used to compare morphological, clinical, demographic, and cognitive indices, as we expected patients to differ from siblings as well as healthy subjects in these phenotypes, with FDR correction to address inflated type 1 error. For data with non-normal distribution (e.g., percentile data on the accuracy of N-back), we used nonparametric Kruskal–Wallis test for statistical analysis. Chi-square analysis was applied for genetic analysis comparing patients and healthy controls. At last, a multivariate generalized linear model with the subgroup based on clusters as the fixed factor was used to test the effect size of all factors including morphological data and phenotypic characteristics.

## Results

### Demographic, genetic, cognitive, and clinical characteristics of all participants

The sequencing of target gene was conducted in a total of 299 participants (135 SZ, 122 HC, and 42 Siblings), of which 31 participants failed to complete clinical data acquisition and MRI scanning, due to withdrawal of consent for specific procedures or contraindications to or poor quality of MRI acquisition. Finally, 268 participants (114 SZ, 112 HC, and 42 Siblings) completed gene sequencing, general information recording, clinical and cognitive assessment, and MRI scanning (Table 1, with a subset of 43 patients providing follow-up symptom and social functioning scores for prognostic assessment). Significant differences were found in age (*P* < 0.001) among the three groups. As expected, patients showed significant impairment in all cognitive tasks and social function compared with HC (Table 1). In addition, the siblings also showed significant impairment in all cognitive tasks and social function compared with HC, but had relatively good social function (*P* < 0.001) and better performance in WCST (*P =* 0.02 for WCST correct, 0.01 for WCST error) and verbal-fluency test (*P =* 0.001) compared with schizophrenia.

**Table 1.** General information, cognitive performance, genetic information, and clinical characteristics of the participants.

	SZ	HC	Sibling	F/ X^2^	*p* value
*General information*	*N* = 114	*N* = 112	*N* = 42		
Age	17.53 ± 2.88	21.73 ± 3.90	22.21 ± 6.07	20.49	<0.001
Sex (male%)	51.75%	51.79%	33.3%	4.82	0.090
WAIS-digital symbol test	63.97 ± 17.84	87.88 ± 16.73	86.48 ± 15.58	57.20	<0.001
WAIS-digit span test (forward)	11.28 ± 2.48	12.01 ± 1.80	11.65 ± 2.01	2.83	0.061
WAIS-digit span test (backward)	6.40 ± 2.64	8.94 ± 3.02	7.53 ± 2.49	21.15	<0.001
WAIS-arithmetic test	14.00 ± 4.290	17.34 ± 3.85	15.70 ± 4.22	16.42	<0.001
SOFAS	61.72 ± 15.11	93.55 ± 4.27	91.15 ± 6.74	241.10	<0.001
eTIV	1.52E + 6 ± 1.49E + 5	1.55E + 6 ± 1.55E + 5	1.55E + 6 ± 1.72E + 5	1.79	0.170
*Cognitive performance*	*N* = 114	*N* = 89	*N* = 40		
Memory-instantaneous story retelling	5.40 ± 2.85	7.55 ± 3.03	5.76 ± 2.51	14.03	<0.001
Memory-short-term story retelling	3.40 ± 2.60	5.73 ± 2.87	3.68 ± 2.91	18.20	<0.001
Memory-N-back component	−0.29 ±1.09	0.38±0.44	−0.09 ± 1.34	11.86	<0.001
Language-verbal-fluency test	17.65 ± 5.38	21.97 ± 6.27	20.13 ± 4.96	14.56	<0.001
Visual perception-visual pattern test (total score)	17.83 ± 4.82	21.27 ± 5.79	18.55 ± 3.98	11.86	<0.001
Visual perception-visual pattern test (max difficulty)	7.25 ± 2.35	9.96 ± 3.70	7.33 ± 2.22	24.40	<0.001
Wisconsin card sorting test (correct)	29.66 ±8.74	37.92 ± 6.28	34.97 ± 8.54	27.22	<0.001
Wisconsin card sorting test (error)	18.41 ±8.31	10.08 ± 6.24	13.06 ± 8.52	29.36	<0.001
*Genetic information*	*N* = 135	*N* = 122	*N* = 42	SZ vs HC	
GRIN2A-rs9940680 (C/G, allele model)	129/141	94/150	44/40	4.468	0.0345
GRIN2A-rs1420040 (G/A, allele model)	126/138	95/149	45/39	3.989	0.0458
GRM3-rs145139281 (GT/GG, dominant model)	10/121	19/103	1/40	3.924	0.0476
CACNA1C-rs73042126 (AA/AG+GG, recessive model)	0/135	4/118	1/41	4.496	0.0340
CACNA1C-Link1_rs10491965_10774044_12578811 (TCAGAG+TTAAAA/CCGGGG, dominant model)	32/103	16/106	7/35	4.731	0.0296
CACNA1C-Link2_rs2239078_74059849 (AG/GT, allele model)	37/233	20/224	8/76	3.942	0.0471
CACNA1C-rs2239016 (AA/AG+GG, recessive model)	15/120	26/96	6/36	4.973	0.0258
CACNA1C-rs57906526 (G/A, allele model)	32/238	45/199	18/66	4.371	0.0366
CACNA1C-rs556844413 (A-/AA, genotype model)	18/117	28/94	8/34	4.034	0.0446
GRIA1-rs2195450(G/A, allele model)	40/226	26/218	5/79	2.169	0.1408
GRIA1-rs3828595(G/T, allele model)	39/231	26/218	5/79	1.665	0.1969
*Clinical information*	*N* = 114				
Age of onset	16.57 ± 3.02				
Duration of untreated psychosis (days)	270 ± 456				
Duration of illness (months)	15.90 ± 16.66				
Duration of medication (months)	7.10 ± 9.63				
Daily chlorpromazine dose equivalents (×100 mg/d)	2.22 ± 1.52				
PANSS (total score)	72.75 ± 24.02				
SAPS (total score)	29.49 ± 19.38				
SANS (total score)	41.73 ±30.04				

As shown in Table 1, association analysis in target genes showed 12 candidate SNPs were different between schizophrenia patients and healthy controls. These SNPs were located in GRIN2A, GRM3, and CACNA1C, but contrary to our expectations, GRIA1 did not differ between patients and control subjects. The differences between the siblings and HC were seen in GRM3 and CACNA1C, and the differences between the siblings and schizophrenia were limited to CACNA1C. Within 12 SNPs, there were two complete linkages between multiple loci (Link1_rs10491965_10774044_12578811 and Link2_rs2239078_ 74059849). Thus, we obtained 9 candidate SNVs with a diagnostic effect for further investigation.

Across the three groups, a general linear model (GLM) with age and sex as covariates showed no significant difference in gyrification among three groups, with minimal differences in cortical thickness, supporting our expectation of morphometric patterns being driven by smaller subgroups of patients (Supplementary Table S1).

### Gaussian clustering and GAP statistics

**Figure 1.**
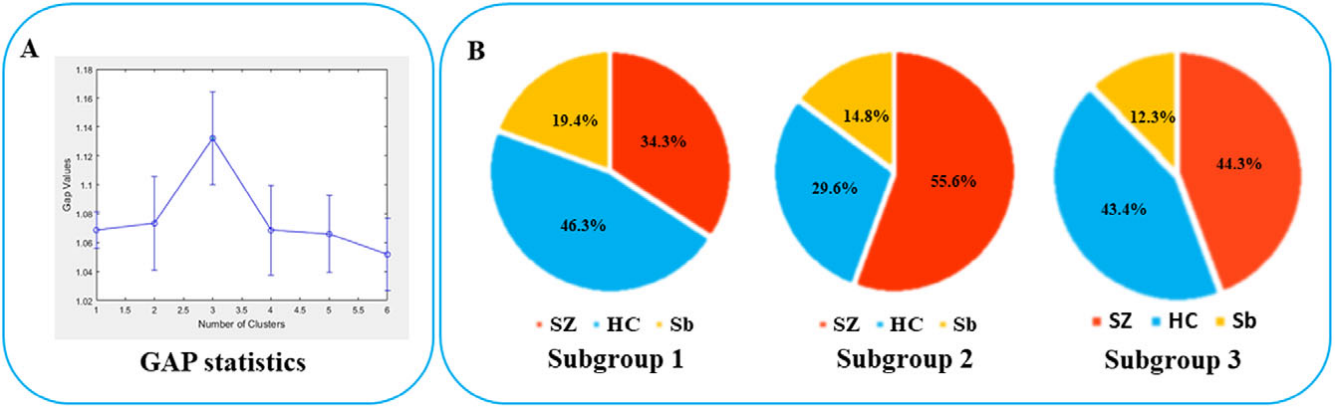
GAP statistics of the clustering by GMM and the composition of each subgroup. (A) GAP statistics when the cluster number was set from 1 to 6; (B) Percentage of components in each of the three subgroups. Notes: GMM, Gaussian mixture model; HC, healthy controls.

We explored the possibility of the existence of one to six clusters by *gmdistribution* (GMM) clustering in Matlab platform, and identified the ideal cluster solution based on GAP statistics to be 3 subgroups (Figure 1A). The numbers of participants composing each subtype were respectively 108 (N_SZ/HC/Sb_=37/50/21), 54(N_SZ/HC/Sb_=30/16/8), and 106 (N_SZ/HC/Sb_= 47/46/13). There are significant differences in the proportion of patients among the three subgroups (*P =* 0.032). Subgroup 2 (*n* = 54) which was the smallest of all three subgroups, had a higher (*P =* 0.009) proportion of patients (55.6% (30/54), *n* = 30) than subgroup 1 (34.3% (37/108), *n* = 37), with subgroup 3 (44.3% (47/106), *n* = 47) being intermediate (Figure 1B). Subgroup 2 also had the lowest proportion of healthy control subjects (Figure 1B). The proportion of siblings in the three subgroups, respectively were 19.4% (*n* = 21), 14.8% (*n* = 8), and 12.3% (*n* = 13), with most siblings clustering together as part of subgroup 1 (50% of all siblings). Most healthy controls also clustered as part of subgroup 1 (41% of all healthy controls).

When comparing patients based on their subgroup membership (Supplementary Table S2), those in subgroup 1 had notable hypo-gyrification in widespread regions compared to subgroups 2 and 3, but there were no significant differences between subgroup 2 and 3 (i.e., gyrification trend: subgroup 1< subgroup 3= subgroup 2). With respect to cortical thickness, subgroup 2 had the highest, subgroup 3 the lowest, and subgroup 1 intermediate values across widespread cortical regions (i.e., thickness trend: subgroup 3< subgroup 1< subgroup 2) (Supplementary Table S2).

**Figure 2.**
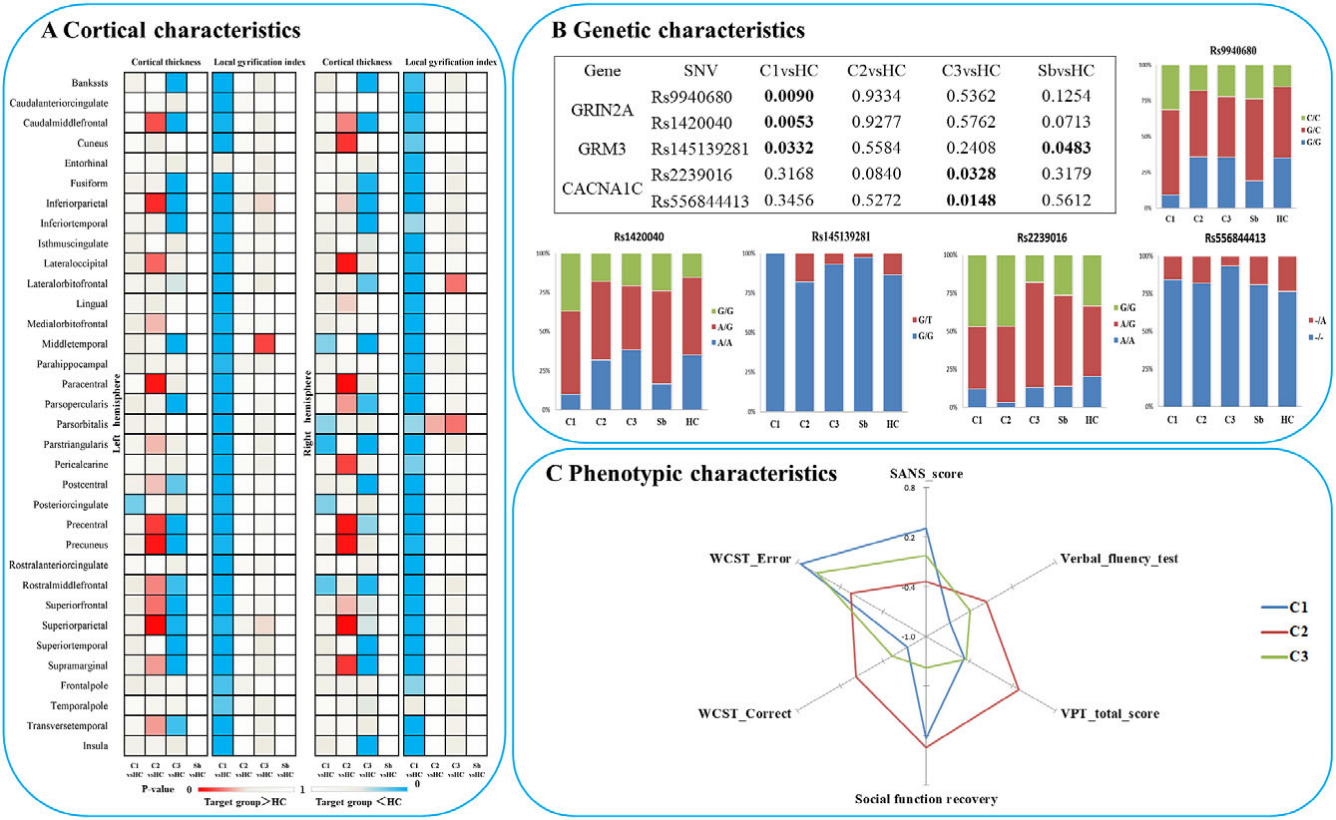
Characteristics of three patient clusters in morphology, clinic, cognition and candidate SNVs. (A) Differences between each subgroup and healthy controls in regional cortical thickness and gyrification; (B) Differences between each subgroup and healthy controls in candidate SNPs; (C) Phenotypic characteristics of each subgroup. Notes: C1, patient cluster in subgroup 1; C2, patient cluster in subgroup 2; C3, patient cluster in subgroup 3; HC, healthy controls; WCST, Wisconsin card sorting test.

When comparing patients based on their subgroup membership against all healthy controls as one group, patients from subgroup 1 displayed a generalized “hypogyric” pattern of widespread reduction in gyrification compared with HCs. Subgroup 2 patients had “supra-normal” pattern, characterized by regional increase in cortical thickness and gyrification. The subgroup 3 patients displayed an “impoverished cortex” pattern with regional cortical thinning compared with HCs (Figure 2A).

### Characteristics of the three patient subgroups in clinical symptoms and cognition

Among the three morphological patient subgroups, there were no significant differences in age and sex. There was no significant difference among the three clusters in estimated total intracranial volume (eTIV), SOFAS, WAIS-Digital symbol test, WAIS-Digit span test (Forward), WAIS-Digit span test (Backward), WAIS-Arithmetic test, N-back, story retelling, onset age, DUP, DoI, DoM, DDD, total PANSS, SAPS, and SRSS, after controlling for the effect of age and sex. However, SANS scores (*P =* 0.014; hypogyric > supra-normal *P =* 0.004) and other cognitive tasks, including verbal-fluency test (*P =* 0.02, hypogyric < supra-normal *P =* 0.005), visual pattern test (total score, *P =* 0.001), Wisconsin Card Sorting Test (WCST correct responses, *P =* 0.013 and errors, *P =* 0.011) varied as per subgroup membership (Figure 2C). Compared to the “hypogyric” subgroup (cluster 1) and “impoverished cortex” subgroups (cluster 3), the “supra-normal” subgroup (cluster 2) also had better visual pattern test performance (*P =* 0.002, 0.001), and WCST performance (more correct (*P =* 0.004, 0.027) and less error (*P =* 0.002, 0.001) responses).

**Figure 3.**
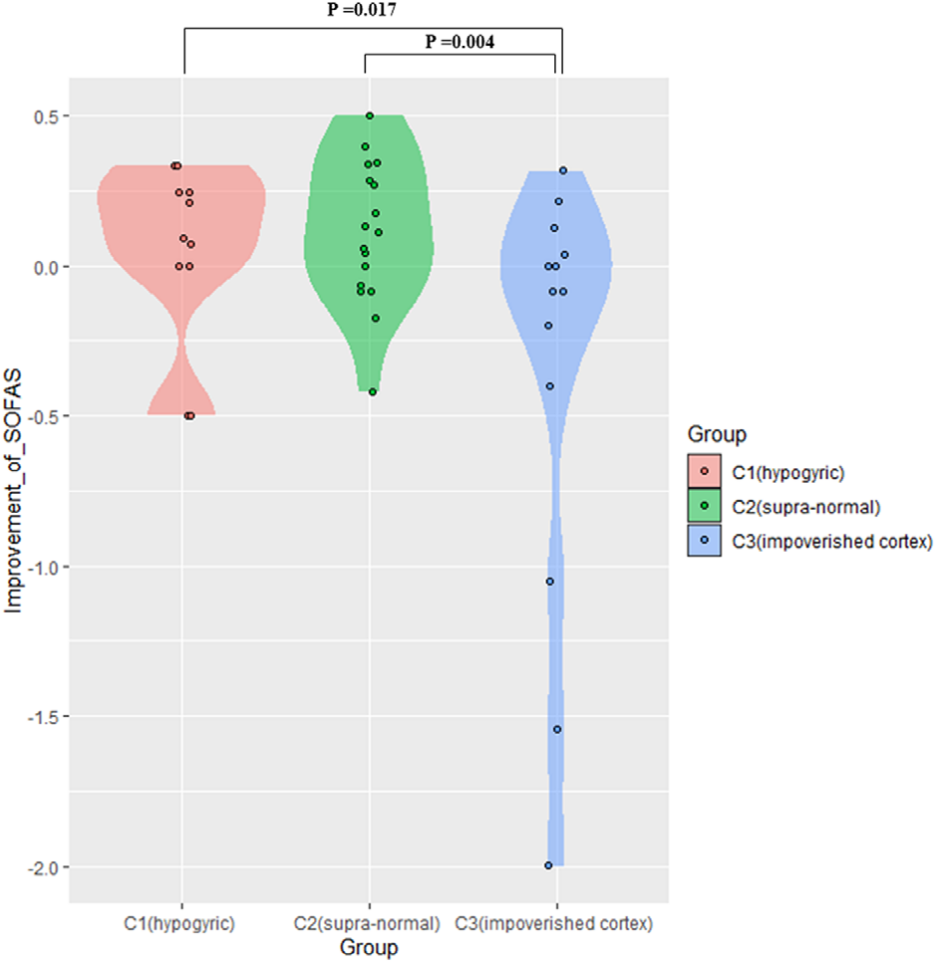
Difference in improvement rate of SOFAS among three patient subgroups. C1, patient cluster in subgroup 1; C2, patient cluster in subgroup 2; C3, patient cluster in subgroup 3.

In the patients with prognostic data (*n* = 43), a GLM with age and sex as covariates did not find significant effects on follow-up time, symptom reduction rate (PANSS, SAPS, SANS) and the change of all cognitive tasks, but there was a significant difference (*P =* 0.01) in social function recovery (improvement rate in SOFAS) among the three clusters. The functional recovery of subgroup 3 (“impoverished cortex,” SOFAS change: mean%[SD]= –62%[119], negative sign indicating deterioration over time) was significantly worse than that of the cluster 1 (“hypogyric,” SOFAS improvement = 5%±30%) (*P =* 0.017) and cluster 2 (“supra-normal,” SOFAS improvement = 13%±25%) (*P =* 0.004) (Figure 3). Taken together, these results indicated that subgroup 1 was the most hypogyric, cognitively affected group with higher negative symptom burden; subgroup 3 had the most reduction in thickness, with most sluggish functional recovery patterns while the subgroup 2 with supra-normal morphometric indices had the best cognitive, symptomatic and functional recovery patterns.

### Differences in candidate SNVs between the subgroups

**Figure 4.**
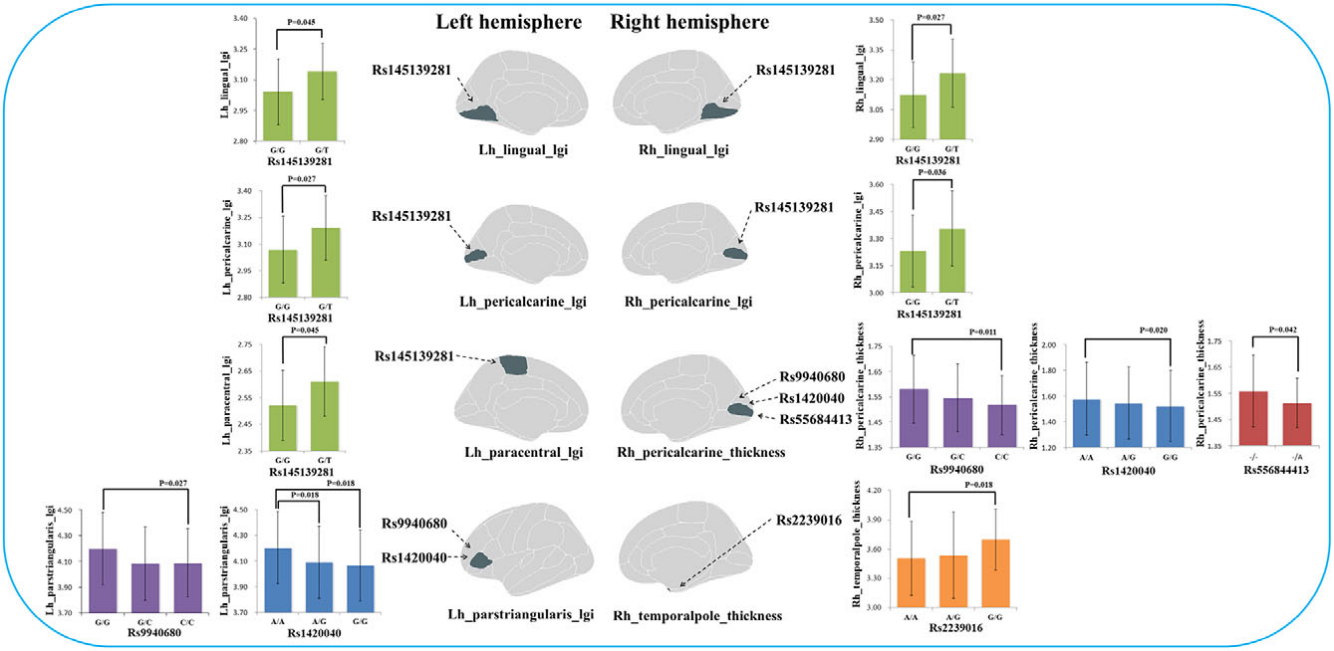
The significant effects of candidate SNVs with differences between subgroups on cortical thickness and gyrification in each significant region. lgi: local gyrification index.

In terms of genetic differences, five SNVs within nine candidate SNVs were identified by the comparisons between each heterogeneous cluster and healthy controls (Figure 2B). The prevalence of variants of rs9940680 (*P =* 0.009) and rs145139281 (*P =* 0.033) were higher in the hypogyric subgroup compared to healthy controls. The rate of prevalence of risk variants in the supra-normal subgroup was not significantly different compared to healthy controls. The prevalence of variants in the impoverished cortex subgroup (cluster 3) was significantly lower in rs223906 (*P =* 0.033) and rs556844413 (*P =* 0.015) compared to healthy controls (Figure 2B). These 5 SNVs with subgroup-level differences were included as candidate SNVs in the following analysis exploring the main effect on morphology (Figure 4) and the interaction between SNVs and morphology (Supplementary Figure S2).

### The effect of candidate SNVs with differences between the subgroups on cortical thickness and gyrification

In all participants, Glm with age and sex as covariates showed that five candidate SNPs with effects on heterogeneous groups (Figure 2B) were also associated with regional cortical thickness and gyrification. Concretely, on cortical gyrification, the effects of rs145139281 (in GRM3) were observed in bilateral lingual (*P*_FDR_ = 0.045, 0.027), bilateral pericalcarine (*P*_FDR_ = 0.027, 0.036) and left paracentral (*P*_FDR_ = 0.045); the effects of rs9940680 and rs1420040 (in GRIN2A) were respectively observed in parstriangularis (*P*_FDR_ = 0.027, 0.018) (Figure 4). On cortical thickness, the effect of rs55684413 (in CACNA1C) was observed in left pericalcarine (*P*_FDR_ = 0.042); the effect of rs2239016 (in CACNA1C) was observed in right temporal pole (*P*_FDR_ = 0.018); the effects of rs9940680 and rs1420040 (in GRIN2A) were also respectively observed in left pericalcarine (*P*_FDR_ = 0.011, 0.02)(Figure 4).

## Discussion

Using a data-driven approach that leverages normal, illness-related as well as shared genetic variance in cortical thickness and gyrification in a relatively early stage (<3 years of illness) of schizophrenia, we report three major findings: (1) three morphologically distinguishable clusters of patients are identifiable (reduced gyrification, reduced thickness, and higher thickness subgroups); (2) phenotypically the subgroups are mostly similar but have the varying burden of negative symptoms, cognitive deficits, and functional deterioration, and (3) the subgroup membership influences illness-related variation in glutamatergic receptor polymorphisms. In addition, the unaffected siblings were more often (50% of siblings) clustered with hypogyric patients, but not with patients who had “supranormal” thickness profiles. This indicates that altered thickness profiles may occur in those who experienced overt symptoms. Familial risk per se, while being insufficient to alter cortical thickness, may influence cortical gyrification patterns. Specific illness-related genetic variations (such as glutamate polymorphism) are more likely in patients/siblings with distinct morphological profiles (i.e., hypogyria).

The effort to discover morphologically homogeneous subgroups among schizophrenia has been expanding in recent years [8, 13, 15, 79]. Despite marked methodological differences (scanner type, metric used, inclusion of healthy variance, etc.), many groups are now reporting at least two broadly consistent subgroups. One with impoverished cortex (reduced thickness or generalized cortical gray matter volume reduction), and the other with preserved or increased cortical grey matter tissue [8, 13, 15, 20]^.^ In the current study, we identified a similar solution, with a preserved thickness (cluster 2) group that displayed better cognitive function compared to others, and a subgroup with widespread cortical thinning (cluster 3) in line with our prior work [13, 20] and previous studies [15, 79]. Again, in line with other prior studies [8, 13, 15, 20, 79], we found that the impoverished cortex subgroup had a more severe illness burden that we were able to observe using functioning data from follow-up assessments.

To our knowledge, prior studies have not utilized the variations in gyrification patterns alongside thickness for subtyping schizophrenia. This is somewhat surprising given the differences in genetic origins of these two measures [80], the differing spatial distribution of thickness and gyrification deficits in schizophrenia [81–83], and the lack of concordance in their progressive trajectories among patients [84]. Consistent with these distinctions, we noted a specific, globally hypogyric subgroup of patients who had no concurrent thickness abnormalities in comparison with healthy subjects. But these patients had higher negative symptom burden, reduced verbal fluency, and persistent functional deficits, during the early phase of treatment (i.e., 2 months of antipsychotic treatment). These observations are in line with prior studies linking gyrification defects with negative symptoms and cognition [85–87]. These results indicated that the intrinsic mechanisms of cortical thickness decline and gyrification decline in schizophrenia are likely to be different, and also indicated the existence of different subgroups in three-dimensional topological morphology. In addition, we also observed a negative correlation (*R* = –0.35, *P*_FDR_ = 0.011) between the cortical thickness of the right medial orbitofrontal and cumulative chlorpromazine equivalent of antipsychotics, indicating that higher dose exposure has a limited effect on the overall cortical morphology.

One of the main goals for subtyping schizophrenia subgroups is to pursue the mechanistic origins of this illness. Our findings relating glutamatergic postsynaptic receptor genes (GRIN2A, GRM3) and CACNA1C gene to subgroups provide an interesting lead in this regard. The subgroup of patients with the most preserved cortical morphology showed no alteration in the glutamate receptor or VGCC risk allele distribution. But the hypogyric subgroup exhibited a higher than expected presence of the schizophrenia-risk SNVs of GRIN2A (rs9940680 and rs1420040) and lower rate of the protective variant of GRM3 (s14513928; a lower rate of this protective variant is also notable in healthy siblings). GRIN2A gene is involved in the synthesis of NMDA receptor complex components, which could directly regulate the permeability of receptors [88]. Increased receptor permeability can lead to an increase in the concentration of cations and glutamate in nerve cells, resulting in excitotoxicity [47, 48]. Shifts in Ca^2+^ currents through NMDA receptors can lead to notable neuronal apoptosis [89], which if occurring during early development, can lead to altered gyrification patterns. GRM3 gene was involved in the synthesis of metabolic glutamate receptor subunit 3, which can inhibit cyclic adenosine monophosphate [90] and reduce Ca^2+^ flow through NMDA receptor, and achieve antioxidant and antineurotoxic effects. Interestingly, animal studies indicate that aberrant gyrification may arise from deficits in astroglial support [91], which may also relate to glutamate-mediated toxicity during development [92]. Note that the effect of these genetic variations may differ across brain regions (see Supplementary Figure S1).

The subgroup with the most prominent thickness reduction (“impoverished cortex”) had a significantly different mutation rate in CACNA1C (higher in risk SNV rs223906, lower in protective SNV rs556844413). In previous studies, the CACNA1C was generally known as one of the regulators of Ca^2+^ signaling in the proliferation and survival of neural progenitors [45, 93]. Previous studies found Ca^2+^ signaling plays an important role in glutamate-mediated excitotoxicity [94, 95], which could deduce neuronal apoptosis [89, 96] resulting in the reduction of cortical thickness [44, 45, 97]. Nevertheless, the lack of association between the impoverished thickness subgroup and glutamatergic receptor AMPA/NMDA variations suggests a less direct role for NMDA hypofunction, and a more direct role for aberrant glutamate dynamics [98] in this subgroup. Besides, the characteristics of treatment-resistant patients indeed overlaps with our “impoverished cortex” subgroup, such as more extensive disruption of brain structure [48, 99, 100], worse cognitive performance [99, 101] and poor functional recovery. Previous studies found that patients with Treatment-Resistant Schizophrenia have increased glutamate levels in the anterior cingulate cortex, whereas dopamine synthesis in the striatum does not differ from controls in this subgroup of patients [102, 103]. One predominant model of dopaminergic dysfunction is that it may occur downstream of excitation/inhibition abnormality in the glutamate system [104]. While we do not have striatal positron-emission tomography (PET) data to confirm this, our observations relating to structure and glutamate receptor polymorphisms are broadly consistent with prior evidence implicating glutamatergic/dopaminergic mechanisms in poor outcomes of schizophrenia.

In summary, the regulation of glutamatergic postsynaptic receptor genes and permeability of calcium channels may regulate cell apoptosis and death, affecting brain morphology both during development and in later life. CACNAC1, GRIN2A, and GRM3 have already been targeted in clinical repurposing trials for schizophrenia [105, 106], but these clinical trials also showed heterogeneous outcomes [107]. The presence of a substantial number of patients (~32.5% in this sample) with supra or near-normal cortical morphology and glutamatergic receptor genes argues for the existence of a non-glutamatergic type of schizophrenia with preserved gyrification, explaining, in part, the heterogeneity of clinical trial outcomes.

The current study has several strengths (multimetric clustering, inclusion of siblings, cluster-based genetic association analysis, and follow-up sample to track functioning); nevertheless, several limitations should also be considered. First, despite a deep sequencing of the target region, we only studied selected genetic locus that we could link a priori with schizophrenia susceptibility and glutamate hypothesis; thus, we did not include all glutamatergic postsynaptic receptor genes. We cannot exclude the regulatory effect of other genes on targeted genes. Second, we lacked transcriptome data to further understand the intermediate process of glutamatergic postsynaptic receptor genes affecting brain morphology. Third, we had limited longitudinal data to study long-term prognostic associations.

To conclude, by linking MRI-derived cortical morphological patterns to glutamatergic and calcium channel variations, we highlight the potential to select patients with certain neuroanatomical features when studying interventions that regulate glutamate/calcium channels in schizophrenia.

## Supplementary material

For supplementary material accompanying this paper visit http://doi.org/10.1192/j.eurpsy.2023.2408. click here to view supplementary material
