# Supplementary material for: Cortical morphological heterogeneity of schizophrenia and its relationship with glutamatergic receptor variations
Source: Eur Psychiatry. 2023 May 9;66(1):e38. doi: 10.1192/j.eurpsy.2023.2408 (PMC10304990; doi:10.1192/j.eurpsy.2023.2408)
Supplement: Supplementary file 1 [file S0924933823024082sup001.docx]

Contents

[Supplement Material A. Bioinformatics analysis of genes 1](#_Toc110585537)

[Supplement Table 1. The effects of diagnosis groups on cortical thickness and gyrification 2](#_Toc110585538)

[Supplement Table 2. The differences in cortical thickness and gyrification among clustering subgroups 4](#_Toc110585539)

[Supplement Figure 1. The effect of candidate SNVs related to heterogeneity on cortical thickness and gyrification 6](#_Toc110585540)

[Supplement Figure 2. The interaction effect between SNVs and heterogeneous groups on cortical thickness and gyrification 6](#_Toc110585541)

[Supplement Material B. The observations in siblings 7](#_Toc110585542)

## Supplement Material A. Bioinformatics analysis of genes

**Alignment**. Raw sequencing data were demultiplexed into individual Fastq read files with Illumina’s bcl2fastq v2.1 6.0.10 based on unique index pairs. Low quality (Q<15) reads/bases were trimmed using fastx tool（http://hannonlab.cshl.edu/fastx_toolkit/index.html by Hannon Lab）. High quality reads were aligned to the NCBI human reference genome (hg19) using the Burrows Wheeler Aligner (BWA) software[[1](#_ENREF_1)]. Subsequently, the aligned reads were processed further using Picard’s MarkDuplicates, SAMtools[[2](#_ENREF_2)] and the Indel Realignment and Base Quality Score Recalibration tools from Genome Analysis Toolkit[[3](#_ENREF_3)].

**Variant calling.** GATK (v3.5)[[3](#_ENREF_3)] and Varscan programs（v2.3.9[[4](#_ENREF_4)] was used to generate genotype information of candidate SNV in targeted regions for each individual, and the called SNV data were then combined. When we called the variants if SNVs had minimal depth coverage >20× and a quality score >30 in more than 80% of the subjects sequenced, a total of 1588 variants within 4 genes were identified.

**Statistical Methods**. Hardy–Weinberg equilibrium for each marker was tested by using the Pearson’s chi-square test with 1 degree of freedom in control populations. Case–control association was tested on the combined case samples as well as individually for work and no-cure groups using chi-square test. All association testing was performed using PLINK version 1.07 ([http://pngu.mgh.harvard.edu/ purcell/plink/](http://pngu.mgh.harvard.edu/%20purcell/plink/))[[5](#_ENREF_5)]. Logistic regression analysis under an additive genetic model was also used to test for the association, adjusting age and sex. For each gene, it compares the number of individuals with no or ≥1 mutations in the gene region between affected and unaffected individuals, using a standard χ2 or Fisher exact test. In this study, we use the χ2 test throughout to avoid bias due to distributional approximation.

## Supplement Table 1. The effects of diagnosis groups on cortical thickness and gyrification

Glm with age and sex as covariates was conducted to analysis the difference in cortical thickness and gyrification. The results showed no significant difference in gyrification among 3 groups (SZ, HC and Sb). But significant differences of cortical thickness were observed in lh_middletemporal, lh_parsopercularis, rh_bankssts, rh_middletemporal, rh_parstriangularis, rh_postcentral and rh_insula. In these regions, schizophrenia showed significant lower thickness compared to healthy control.

| Supplement table 1. The effects of diagnosis groups on cortical thickness and gyrification | | | | | | | |
| --- | --- | --- | --- | --- | --- | --- | --- |
| Gyrification of each region | F | Partial Eta^2^ | P_FDR | Cortical thickness of each region | F | Partial Eta2 | P_FDR |
| lh_bankssts_lgi | .971 | .007 | 0.834 | lh_bankssts_thickness | 4.530 | .033 | 0.079 |
| lh_caudalanteriorcingulate_lgi | 2.418 | .018 | 0.680 | lh_caudalanteriorcingulate_thickness | .515 | .004 | 0.697 |
| lh_caudalmiddlefrontal_lgi | 1.845 | .014 | 0.680 | lh_caudalmiddlefrontal_thickness | 1.843 | .014 | 0.328 |
| lh_cuneus_lgi | 2.025 | .015 | 0.680 | lh_cuneus_thickness | .017 | .000 | 0.983 |
| lh_entorhinal_lgi | .345 | .003 | 0.852 | lh_entorhinal_thickness | 1.137 | .009 | 0.492 |
| lh_fusiform_lgi | .307 | .002 | 0.863 | lh_fusiform_thickness | 4.311 | .032 | 0.079 |
| lh_inferiorparietal_lgi | .508 | .004 | 0.852 | lh_inferiorparietal_thickness | 2.773 | .021 | 0.218 |
| lh_inferiortemporal_lgi | .023 | .000 | 0.977 | lh_inferiortemporal_thickness | 4.356 | .032 | 0.079 |
| lh_isthmuscingulate_lgi | 1.007 | .008 | 0.834 | lh_isthmuscingulate_thickness | 2.418 | .018 | 0.238 |
| lh_lateraloccipital_lgi | 1.648 | .012 | 0.697 | lh_lateraloccipital_thickness | 1.074 | .008 | 0.492 |
| lh_lateralorbitofrontal_lgi | .738 | .006 | 0.852 | lh_lateralorbitofrontal_thickness | 1.166 | .009 | 0.492 |
| lh_lingual_lgi | 1.013 | .008 | 0.834 | lh_lingual_thickness | .635 | .005 | 0.657 |
| lh_medialorbitofrontal_lgi | 1.194 | .009 | 0.830 | lh_medialorbitofrontal_thickness | .998 | .008 | 0.503 |
| lh_middletemporal_lgi | .035 | .000 | 0.977 | **lh_middletemporal_thickness** | **6.043** | **.044** | **0.049** |
| lh_parahippocampal_lgi | .545 | .004 | 0.852 | lh_parahippocampal_thickness | 3.110 | .023 | 0.184 |
| lh_paracentral_lgi | 3.803 | .028 | 0.589 | lh_paracentral_thickness | .155 | .001 | 0.896 |
| lh_parsopercularis_lgi | 1.928 | .014 | 0.680 | **lh_parsopercularis_thickness** | **8.002** | **.057** | **0.029** |
| lh_parsorbitalis_lgi | .592 | .004 | 0.852 | lh_parsorbitalis_thickness | 2.969 | .022 | 0.200 |
| lh_parstriangularis_lgi | 1.772 | .013 | 0.688 | lh_parstriangularis_thickness | 1.083 | .008 | 0.492 |
| lh_pericalcarine_lgi | 2.684 | .020 | 0.680 | lh_pericalcarine_thickness | .672 | .005 | 0.656 |
| lh_postcentral_lgi | 3.815 | .028 | 0.589 | lh_postcentral_thickness | 2.057 | .015 | 0.295 |
| lh_posteriorcingulate_lgi | 1.906 | .014 | 0.680 | lh_posteriorcingulate_thickness | 2.183 | .016 | 0.279 |
| lh_precentral_lgi | 3.705 | .027 | 0.589 | lh_precentral_thickness | 1.366 | .010 | 0.436 |
| lh_precuneus_lgi | .369 | .003 | 0.852 | lh_precuneus_thickness | 2.419 | .018 | 0.238 |
| lh_rostralanteriorcingulate_lgi | 1.976 | .015 | 0.680 | lh_rostralanteriorcingulate_thickness | .433 | .003 | 0.723 |
| lh_rostralmiddlefrontal_lgi | 3.397 | .025 | 0.595 | lh_rostralmiddlefrontal_thickness | 2.100 | .016 | 0.293 |
| lh_superiorfrontal_lgi | 1.861 | .014 | 0.680 | lh_superiorfrontal_thickness | 2.517 | .019 | 0.238 |
| lh_superiorparietal_lgi | 1.093 | .008 | 0.834 | lh_superiorparietal_thickness | .486 | .004 | 0.697 |
| lh_superiortemporal_lgi | .621 | .005 | 0.852 | lh_superiortemporal_thickness | 3.463 | .026 | 0.173 |
| lh_supramarginal_lgi | 1.934 | .014 | 0.680 | lh_supramarginal_thickness | 3.293 | .024 | 0.179 |
| lh_frontalpole_lgi | .769 | .006 | 0.852 | lh_frontalpole_thickness | .332 | .003 | 0.780 |
| lh_temporalpole_lgi | .073 | .001 | 0.977 | lh_temporalpole_thickness | 1.438 | .011 | 0.422 |
| lh_transversetemporal_lgi | 1.417 | .011 | 0.773 | lh_transversetemporal_thickness | 1.070 | .008 | 0.492 |
| lh_insula_lgi | .398 | .003 | 0.852 | lh_insula_thickness | 1.344 | .010 | 0.436 |
| rh_bankssts_lgi | .050 | .000 | 0.977 | **rh_bankssts_thickness** | **5.504** | **.040** | **0.049** |
| rh_caudalanteriorcingulate_lgi | 1.394 | .010 | 0.773 | rh_caudalanteriorcingulate_thickness | 1.064 | .008 | 0.492 |
| rh_caudalmiddlefrontal_lgi | .399 | .003 | 0.852 | rh_caudalmiddlefrontal_thickness | 1.796 | .013 | 0.328 |
| rh_cuneus_lgi | .525 | .004 | 0.852 | rh_cuneus_thickness | .076 | .001 | 0.943 |
| rh_entorhinal_lgi | .390 | .003 | 0.852 | rh_entorhinal_thickness | .616 | .005 | 0.657 |
| rh_fusiform_lgi | .241 | .002 | 0.900 | rh_fusiform_thickness | 2.519 | .019 | 0.238 |
| rh_inferiorparietal_lgi | 1.316 | .010 | 0.798 | rh_inferiorparietal_thickness | 1.031 | .008 | 0.497 |
| rh_inferiortemporal_lgi | .437 | .003 | 0.852 | rh_inferiortemporal_thickness | 4.809 | .035 | 0.068 |
| rh_isthmuscingulate_lgi | .034 | .000 | 0.977 | rh_isthmuscingulate_thickness | 2.487 | .019 | 0.238 |
| rh_lateraloccipital_lgi | 2.533 | .019 | 0.680 | rh_lateraloccipital_thickness | .496 | .004 | 0.697 |
| rh_lateralorbitofrontal_lgi | .216 | .002 | 0.900 | rh_lateralorbitofrontal_thickness | 2.891 | .022 | 0.204 |
| rh_lingual_lgi | .585 | .004 | 0.852 | rh_lingual_thickness | .325 | .002 | 0.780 |
| rh_medialorbitofrontal_lgi | .908 | .007 | 0.852 | rh_medialorbitofrontal_thickness | .746 | .006 | 0.621 |
| rh_middletemporal_lgi | .431 | .003 | 0.852 | **rh_middletemporal_thickness** | **6.213** | **.045** | **0.049** |
| rh_parahippocampal_lgi | .449 | .003 | 0.852 | rh_parahippocampal_thickness | 2.266 | .017 | 0.267 |
| rh_paracentral_lgi | .813 | .006 | 0.852 | rh_paracentral_thickness | 1.077 | .008 | 0.492 |
| rh_parsopercularis_lgi | 1.593 | .012 | 0.697 | rh_parsopercularis_thickness | 1.845 | .014 | 0.328 |
| rh_parsorbitalis_lgi | .783 | .006 | 0.852 | rh_parsorbitalis_thickness | 1.789 | .013 | 0.328 |
| rh_parstriangularis_lgi | .540 | .004 | 0.852 | **rh_parstriangularis_thickness** | **5.903** | **.043** | **0.049** |
| rh_pericalcarine_lgi | .214 | .002 | 0.900 | rh_pericalcarine_thickness | 1.645 | .012 | 0.358 |
| rh_postcentral_lgi | 1.629 | .012 | 0.697 | **rh_postcentral_thickness** | **5.712** | **.042** | **0.049** |
| rh_posteriorcingulate_lgi | .748 | .006 | 0.852 | rh_posteriorcingulate_thickness | 3.222 | .024 | 0.179 |
| rh_precentral_lgi | 2.295 | .017 | 0.680 | rh_precentral_thickness | .255 | .002 | 0.823 |
| rh_precuneus_lgi | .099 | .001 | 0.977 | rh_precuneus_thickness | .497 | .004 | 0.697 |
| rh_rostralanteriorcingulate_lgi | .705 | .005 | 0.852 | rh_rostralanteriorcingulate_thickness | .074 | .001 | 0.943 |
| rh_rostralmiddlefrontal_lgi | .154 | .001 | 0.941 | rh_rostralmiddlefrontal_thickness | 3.219 | .024 | 0.179 |
| rh_superiorfrontal_lgi | .338 | .003 | 0.852 | rh_superiorfrontal_thickness | 1.429 | .011 | 0.422 |
| rh_superiorparietal_lgi | 2.452 | .018 | 0.680 | rh_superiorparietal_thickness | .648 | .005 | 0.657 |
| rh_superiortemporal_lgi | .976 | .007 | 0.834 | rh_superiortemporal_thickness | 5.012 | .037 | 0.060 |
| rh_supramarginal_lgi | .571 | .004 | 0.852 | rh_supramarginal_thickness | 2.596 | .019 | 0.238 |
| rh_frontalpole_lgi | .762 | .006 | 0.852 | rh_frontalpole_thickness | .785 | .006 | 0.609 |
| rh_temporalpole_lgi | .489 | .004 | 0.852 | rh_temporalpole_thickness | 1.806 | .014 | 0.328 |
| rh_transversetemporal_lgi | 1.206 | .009 | 0.830 | rh_transversetemporal_thickness | 1.701 | .013 | 0.348 |
| rh_insula_lgi | 1.137 | .009 | 0.834 | **rh_insula_thickness** | **5.459** | **.040** | **0.049** |
| Notes: lh, left hemisphere; rh, right hemisphrere. | | | | | | | |

## Supplement Table 2. The differences in cortical thickness and gyrification among clustering subgroups

After clustering solution was determined, Glm with age and sex as covariates was conducted to reveal the cortical morphological features of each subgroup. As showed in below table, we found subgroup 1 (S1) showed hypo-gyrification in widespread regions compared to subgroup 2 (S2) and subgroup 3 (S3), but there is no significant difference between subgroup 2 and 3. Across three subgroups, the cortical thickness of S2 is the greatest, S3 is the smallest, and S1 is at the intermediate level in widespread regions. From the overall trend, the gyrification of the three subgroups showed the following order: S1< S3= S2, and the cortical thickness showed S3< S1< S2.

| Supplement table 2. The differences in cortical thickness and gyrification among clustering subgroups | | | | | | | |
| --- | --- | --- | --- | --- | --- | --- | --- |
| Gyrification of each region | P_FDR (S1<S3=S2) | | | Cortical thickness of each region | P_FDR (S3<S1<S2) | | |
|  | S1vsS2 | S1vsS3 | S2vsS3 |  | S1vsS2 | S1vsS3 | S2vsS3 |
| lh_bankssts_lgi | 5.26E-11 | 7.82E-21 | .688 | lh_bankssts_thickness | 0.0548 | 0.0000 | 0.0001 |
| lh_caudalanteriorcingulate_lgi | 5.52E-04 | 1.20E-10 | .665 | lh_caudalanteriorcingulate_thickness | 0.0548 | 0.0015 | 0.0300 |
| lh_caudalmiddlefrontal_lgi | 9.22E-06 | 1.53E-11 | .932 | lh_caudalmiddlefrontal_thickness | 0.0001 | 0.0000 | 0.0000 |
| lh_cuneus_lgi | 1.81E-04 | 4.18E-10 | .996 | lh_cuneus_thickness | 0.0416 | 0.0304 | 0.8039 |
| lh_entorhinal_lgi | 4.94E-03 | 4.37E-06 | .715 | lh_entorhinal_thickness | 0.4552 | 0.0018 | 0.0209 |
| lh_fusiform_lgi | 7.19E-07 | 3.38E-17 | .665 | lh_fusiform_thickness | 0.0002 | 0.0000 | 0.0000 |
| lh_inferiorparietal_lgi | 1.90E-12 | 6.92E-21 | .996 | lh_inferiorparietal_thickness | 0.0001 | 0.0000 | 0.0000 |
| lh_inferiortemporal_lgi | 2.95E-06 | 3.58E-14 | .665 | lh_inferiortemporal_thickness | 0.0710 | 0.0000 | 0.0000 |
| lh_isthmuscingulate_lgi | 2.98E-06 | 1.72E-13 | .665 | lh_isthmuscingulate_thickness | 0.0384 | 0.0000 | 0.0336 |
| lh_lateraloccipital_lgi | 8.17E-05 | 2.09E-15 | .692 | lh_lateraloccipital_thickness | 0.0000 | 0.0000 | 0.3076 |
| lh_lateralorbitofrontal_lgi | 3.40E-13 | 4.07E-15 | .715 | lh_lateralorbitofrontal_thickness | 0.0104 | 0.0000 | 0.0143 |
| lh_lingual_lgi | 9.89E-06 | 1.37E-12 | .692 | lh_lingual_thickness | 0.0001 | 0.0001 | 0.7354 |
| lh_medialorbitofrontal_lgi | 2.65E-04 | 3.22E-07 | .996 | lh_medialorbitofrontal_thickness | 0.0002 | 0.0023 | 0.2629 |
| lh_middletemporal_lgi | 6.48E-10 | 1.46E-21 | .370 | lh_middletemporal_thickness | 0.0020 | 0.0000 | 0.0000 |
| lh_parahippocampal_lgi | 5.09E-05 | 7.04E-12 | .682 | lh_parahippocampal_thickness | 0.9530 | 0.1021 | 0.0144 |
| lh_paracentral_lgi | 6.26E-04 | 4.08E-11 | .665 | lh_paracentral_thickness | 0.0000 | 0.0000 | 0.0000 |
| lh_parsopercularis_lgi | 2.39E-09 | 3.43E-16 | .692 | lh_parsopercularis_thickness | 0.0000 | 0.0000 | 0.0025 |
| lh_parsorbitalis_lgi | 4.50E-07 | 6.69E-09 | .715 | lh_parsorbitalis_thickness | 0.0010 | 0.0014 | 0.6421 |
| lh_parstriangularis_lgi | 2.03E-09 | 4.45E-16 | .665 | lh_parstriangularis_thickness | 0.0001 | 0.0000 | 0.1879 |
| lh_pericalcarine_lgi | 7.11E-05 | 4.57E-09 | .989 | lh_pericalcarine_thickness | 0.0047 | 0.2030 | 0.1444 |
| lh_postcentral_lgi | 1.20E-13 | 1.15E-20 | .978 | lh_postcentral_thickness | 0.0001 | 0.0000 | 0.0307 |
| lh_posteriorcingulate_lgi | 1.18E-05 | 7.59E-09 | .996 | lh_posteriorcingulate_thickness | 0.0085 | 0.0006 | 0.4158 |
| lh_precentral_lgi | 1.20E-13 | 1.99E-18 | .758 | lh_precentral_thickness | 0.0000 | 0.0000 | 0.0000 |
| lh_precuneus_lgi | 6.08E-05 | 1.36E-11 | .692 | lh_precuneus_thickness | 0.0002 | 0.0000 | 0.0000 |
| lh_rostralanteriorcingulate_lgi | 1.96E-04 | 5.14E-09 | .896 | lh_rostralanteriorcingulate_thickness | 0.9530 | 0.0552 | 0.0209 |
| lh_rostralmiddlefrontal_lgi | 7.38E-07 | 1.42E-13 | .974 | lh_rostralmiddlefrontal_thickness | 0.0000 | 0.0000 | 0.1110 |
| lh_superiorfrontal_lgi | 5.09E-05 | 3.94E-16 | .370 | lh_superiorfrontal_thickness | 0.0000 | 0.0000 | 0.0000 |
| lh_superiorparietal_lgi | 2.07E-07 | 4.45E-16 | .715 | lh_superiorparietal_thickness | 0.0000 | 0.0000 | 0.0000 |
| lh_superiortemporal_lgi | 8.31E-12 | 1.45E-28 | .068 | lh_superiortemporal_thickness | 0.1625 | 0.0000 | 0.0001 |
| lh_supramarginal_lgi | 4.72E-11 | 2.01E-18 | .715 | lh_supramarginal_thickness | 0.0001 | 0.0000 | 0.0000 |
| lh_frontalpole_lgi | 1.42E-02 | 1.44E-05 | .665 | lh_frontalpole_thickness | 0.0003 | 0.0001 | 0.3792 |
| lh_temporalpole_lgi | 8.15E-03 | 3.49E-07 | .704 | lh_temporalpole_thickness | 0.4102 | 0.1817 | 0.0459 |
| lh_transversetemporal_lgi | 2.57E-11 | 3.25E-25 | .306 | lh_transversetemporal_thickness | 0.0186 | 0.0000 | 0.0000 |
| lh_insula_lgi | 8.37E-11 | 5.43E-19 | .370 | lh_insula_thickness | 0.0167 | 0.0000 | 0.0478 |
| rh_bankssts_lgi | 3.02E-08 | 2.56E-12 | .978 | rh_bankssts_thickness | 0.0841 | 0.0000 | 0.0002 |
| rh_caudalanteriorcingulate_lgi | 1.30E-05 | 8.56E-11 | .665 | rh_caudalanteriorcingulate_thickness | 0.0566 | 0.1246 | 0.6631 |
| rh_caudalmiddlefrontal_lgi | 3.07E-05 | 6.48E-07 | .843 | rh_caudalmiddlefrontal_thickness | 0.0018 | 0.0000 | 0.0000 |
| rh_cuneus_lgi | 3.07E-05 | 1.07E-11 | .866 | rh_cuneus_thickness | 0.0000 | 0.0000 | 0.4358 |
| rh_entorhinal_lgi | 1.51E-02 | 1.26E-09 | .370 | rh_entorhinal_thickness | 0.7788 | 0.0023 | 0.0023 |
| rh_fusiform_lgi | 1.84E-06 | 1.96E-15 | .428 | rh_fusiform_thickness | 0.0076 | 0.0000 | 0.0000 |
| rh_inferiorparietal_lgi | 1.93E-12 | 2.67E-19 | .715 | rh_inferiorparietal_thickness | 0.0014 | 0.0000 | 0.0000 |
| rh_inferiortemporal_lgi | 9.68E-05 | 9.55E-11 | .665 | rh_inferiortemporal_thickness | 0.0533 | 0.0000 | 0.0000 |
| rh_isthmuscingulate_lgi | 2.67E-05 | 1.70E-12 | .665 | rh_isthmuscingulate_thickness | 0.0166 | 0.0004 | 0.2534 |
| rh_lateraloccipital_lgi | 4.15E-06 | 2.23E-13 | .715 | rh_lateraloccipital_thickness | 0.0000 | 0.0000 | 0.0478 |
| rh_lateralorbitofrontal_lgi | 1.58E-11 | 3.00E-19 | .665 | rh_lateralorbitofrontal_thickness | 0.0046 | 0.0000 | 0.0640 |
| rh_lingual_lgi | 4.01E-05 | 1.36E-13 | .665 | rh_lingual_thickness | 0.0000 | 0.0001 | 0.8039 |
| rh_medialorbitofrontal_lgi | 2.35E-08 | 6.87E-14 | .692 | rh_medialorbitofrontal_thickness | 0.0680 | 0.0317 | 0.8039 |
| rh_middletemporal_lgi | 5.61E-07 | 2.23E-13 | .665 | rh_middletemporal_thickness | 0.0205 | 0.0000 | 0.0009 |
| rh_parahippocampal_lgi | 5.98E-04 | 7.36E-11 | .370 | rh_parahippocampal_thickness | 0.8966 | 0.0718 | 0.0129 |
| rh_paracentral_lgi | 8.28E-05 | 1.70E-12 | .715 | rh_paracentral_thickness | 0.0000 | 0.0000 | 0.0006 |
| rh_parsopercularis_lgi | 2.99E-10 | 1.09E-17 | .665 | rh_parsopercularis_thickness | 0.0058 | 0.0000 | 0.0002 |
| rh_parsorbitalis_lgi | 1.66E-08 | 5.34E-08 | .692 | rh_parsorbitalis_thickness | 0.0014 | 0.0001 | 0.3792 |
| rh_parstriangularis_lgi | 8.50E-09 | 8.50E-18 | .665 | rh_parstriangularis_thickness | 0.0157 | 0.0000 | 0.1689 |
| rh_pericalcarine_lgi | 8.17E-05 | 1.48E-12 | .692 | rh_pericalcarine_thickness | 0.0022 | 0.0511 | 0.3627 |
| rh_postcentral_lgi | 3.84E-15 | 6.65E-19 | .704 | rh_postcentral_thickness | 0.0243 | 0.0000 | 0.0006 |
| rh_posteriorcingulate_lgi | 5.26E-05 | 1.56E-11 | .665 | rh_posteriorcingulate_thickness | 0.0002 | 0.0000 | 0.7354 |
| rh_precentral_lgi | 2.99E-14 | 2.67E-19 | .848 | rh_precentral_thickness | 0.0000 | 0.0000 | 0.0002 |
| rh_precuneus_lgi | 1.48E-07 | 7.53E-14 | .842 | rh_precuneus_thickness | 0.0001 | 0.0000 | 0.0002 |
| rh_rostralanteriorcingulate_lgi | 1.48E-07 | 6.00E-13 | .715 | rh_rostralanteriorcingulate_thickness | 0.9530 | 0.1351 | 0.0384 |
| rh_rostralmiddlefrontal_lgi | 2.39E-09 | 7.63E-17 | .665 | rh_rostralmiddlefrontal_thickness | 0.0000 | 0.0000 | 0.0449 |
| rh_superiorfrontal_lgi | 6.08E-05 | 3.72E-14 | .370 | rh_superiorfrontal_thickness | 0.0001 | 0.0000 | 0.0011 |
| rh_superiorparietal_lgi | 4.72E-11 | 7.63E-17 | .692 | rh_superiorparietal_thickness | 0.0000 | 0.0000 | 0.0001 |
| rh_superiortemporal_lgi | 9.08E-10 | 2.00E-19 | .370 | rh_superiortemporal_thickness | 0.0680 | 0.0000 | 0.0011 |
| rh_supramarginal_lgi | 1.72E-14 | 4.26E-17 | .793 | rh_supramarginal_thickness | 0.0024 | 0.0000 | 0.0000 |
| rh_frontalpole_lgi | 1.96E-04 | 4.38E-08 | .665 | rh_frontalpole_thickness | 0.0157 | 0.0304 | 0.8340 |
| rh_temporalpole_lgi | 1.56E-04 | 5.87E-10 | .665 | rh_temporalpole_thickness | 0.5213 | 0.0344 | 0.0015 |
| rh_transversetemporal_lgi | 1.87E-10 | 1.69E-18 | .665 | rh_transversetemporal_thickness | 0.1313 | 0.0000 | 0.0044 |
| rh_insula_lgi | 3.05E-11 | 2.00E-19 | .665 | rh_insula_thickness | 0.1881 | 0.0002 | 0.0021 |
| Notes: lh, left hemisphere; rh, right hemisphrere. | | | | | | | |

## Supplement Figure 1. The effect of candidate SNVs related to heterogeneity on cortical thickness and gyrification

In all participants, Glm with age and sex as covariates showed that 5 candidate SNPs with effects on heterogeneous groups (Figure.2B in manuscript) was also associated with regional cortical thickness and gyrification. Concretely, on cortical gyrification, the effects of rs145139281 were observed in bilateral lingual (P_FDR_=0.045, 0.027), bilateral pericalcarine (P_FDR_=0.027, 0.036) and left paracentral (P_FDR_=0.045); the effects of rs9940680 and rs14200040 were respectively observed in parstriangularis (P_FDR_=0.027, 0.018) (Supplement Figure.1). On cortical thickness, the effect of rs55684413 was observed in left pericalcarine (P_FDR_=0.042); the effect of rs2239016 was observed in right temporal pole (P_FDR_=0.018); the effects of rs9940680 and rs14200040 were also respectively observed in left pericalcarine (P_FDR_=0.011, 0.02) (Supplement Figure.1).

Supplement
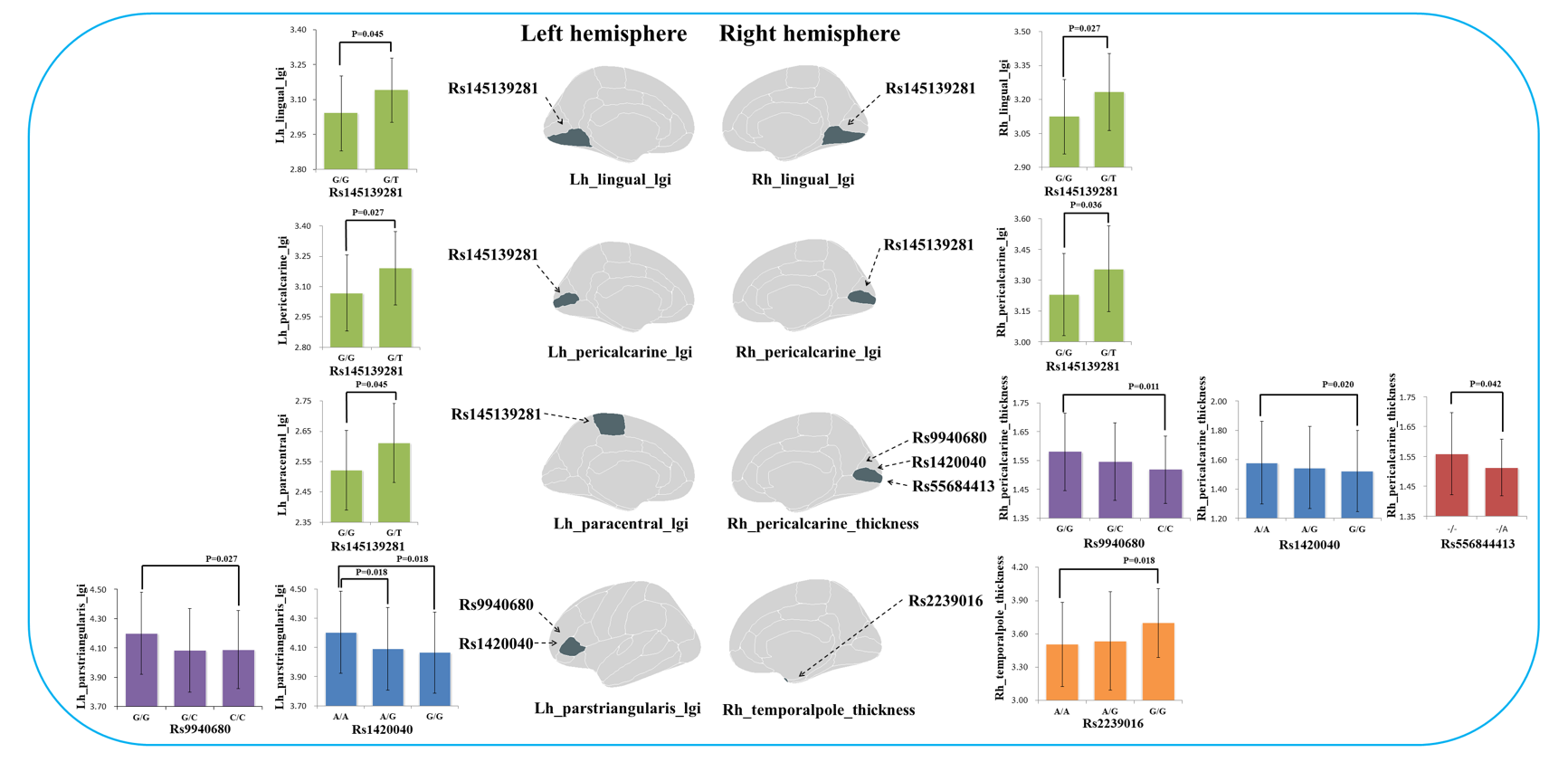
Figure 1. The significant effects of candidate SNVs on cortical thickness and gyrification in each significant region. Note: lgi, the local gyrification index. GRIN2A (rs9940680 and rs1420040) was more closely related to the morphology of left parstriangularis and pericalcarine; GRM3 (rs145139281) was more closely related to the morphology of bilateral lingual, pericalcarine and left paracentral; CACNA1C (rs55684413 and rs2239016) was more closely related to the morphology of left pericalcarine and right temporal pole.

## Supplement Figure 2. The interaction effect between SNVs and heterogeneous groups on cortical thickness and gyrification

Additionally, the current study also found an interaction effect between two risk SNVs (rs9940680, P=0.029 and rs1420040, P=0.011) of GRIN2A and heterogeneous group on cortical gyrification of left parstriangularis, which showed that the effect of risk SNVs in supranormal cluster was similar to healthy controls: the homozygous subjects without polymorphisms (G/G or A/A respectively) exhibited higher gyrification in left parstriangularis than the homozygous subjects with polymorphisms (C/C or G/G). But a similar effect was not observed in hypo-gyric and impoverished-thickness clusters. Thus, the relationship between gyrification in left parstriangularis and SNVs of GRIN2A was restricted to the supranormal group (Supplement Figure.1).


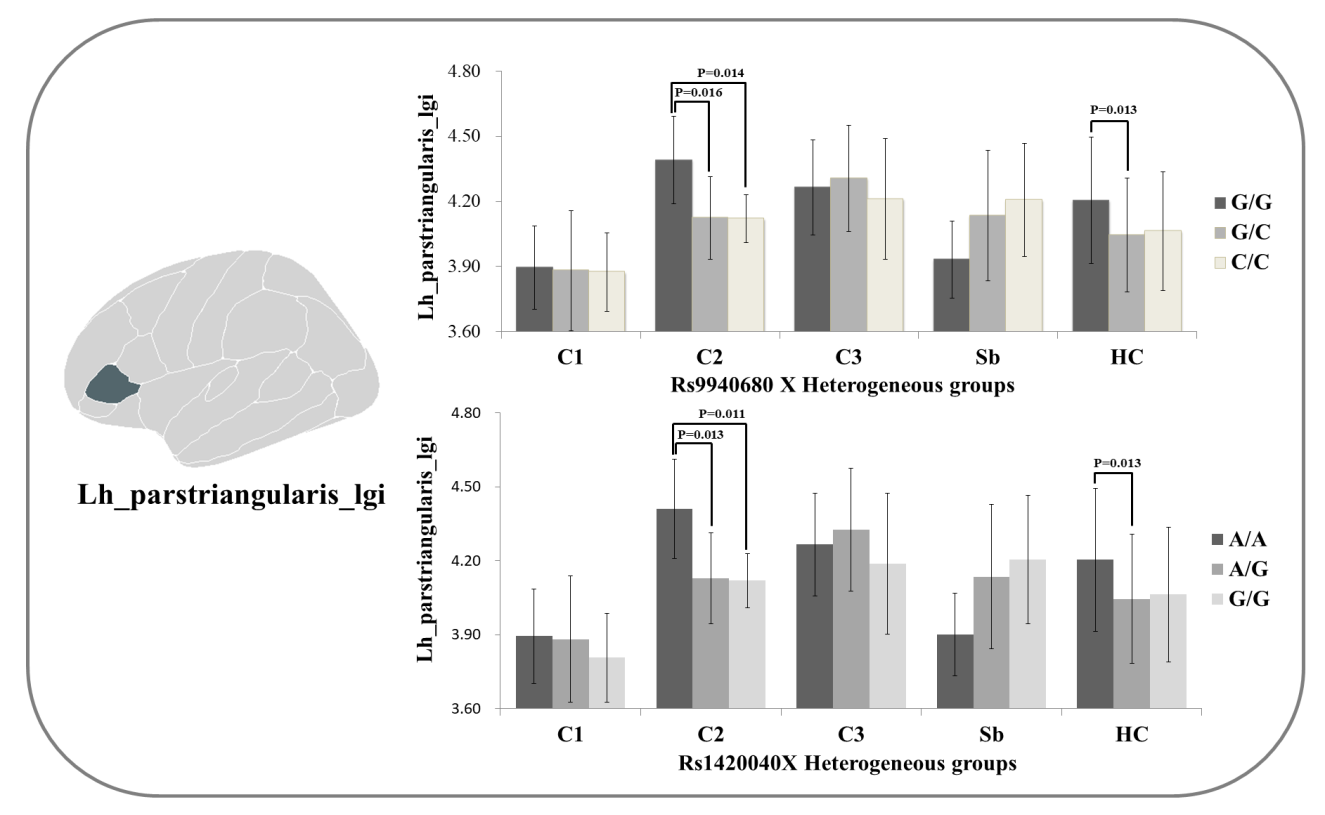


Supplement Figure.2 Interaction effect of rs9940680 and rs1420040 X Heterogeneous group on cortical gyrification of left parstriangularis. A specific role of GRIN2A (rs9940680 and rs1420040) in left inferior frontal – parstriangularis (Broca’s area) morphology was observed.

## Supplement Material B. The observations in siblings

The current study also included the unaffected siblings as a control group. With respect to the cortical morphometric analysis, the unaffected siblings showed no difference from healthy controls, indicating the need for higher genetic dose, or other interacting non-genetic factors in shaping cortical morphology. But the cognitive functioning of siblings was intermediate between schizophrenia and healthy group, consistent with previous studies(6-9). The siblings also showed lower variation rate in protective SNV (rs14513928) of GRM3 compared to healthy controls. The interaction between gyrification in left parstriangularis and SNVs of GRIN2A that was seen among patients, was not observed among the siblings.

**Reference**

[1] Li H, Durbin R. Fast and accurate long-read alignment with Burrows-Wheeler transform. Bioinformatics. 2010;26:589-95.

[2] Li H, Handsaker B, Wysoker A, Fennell T, Ruan J, Homer N, et al. The Sequence Alignment/Map format and SAMtools. Bioinformatics. 2009;25:2078-9.

[3] McKenna A, Hanna M, Banks E, Sivachenko A, Cibulskis K, Kernytsky A, et al. The Genome Analysis Toolkit: a MapReduce framework for analyzing next-generation DNA sequencing data. Genome research. 2010;20:1297-303.

[4] Koboldt DC, Zhang Q, Larson DE, Shen D, McLellan MD, Lin L, et al. VarScan 2: somatic mutation and copy number alteration discovery in cancer by exome sequencing. Genome research. 2012;22:568-76.

[5] Purcell S, Neale B, Todd-Brown K, Thomas L, Ferreira MA, Bender D, et al. PLINK: a tool set for whole-genome association and population-based linkage analyses. Am J Hum Genet. 2007;81:559-75.
